# The incretin effect in critically ill patients: a case–control study

**DOI:** 10.1186/s13054-015-1118-z

**Published:** 2015-11-16

**Authors:** Signe Tellerup Nielsen, Susanne Janum, Rikke Krogh-Madsen, Thomas P. Solomon, Kirsten Møller

**Affiliations:** Centre of Inflammation and Metabolism and the Centre for Physical Activity Research, Rigshospitalet, University of Copenhagen, Copenhagen, Denmark; Department of Anaesthesiology, Bispebjerg Hospital, University of Copenhagen, Copenhagen, Denmark; School of Sport, Exercise, and Rehabilitation Sciences, Centre for Endocrinology, Diabetes, and Metabolism, University of Birmingham, Birmingham, UK; Neurointensive Care Unit, Department of Neuroanaesthesiology, Rigshospitalet, University of Copenhagen, Copenhagen, Denmark

## Abstract

**Introduction:**

Patients admitted to the intensive care unit often develop hyperglycaemia, but the underlying mechanisms have not been fully described. The incretin effect is reduced in patients with type 2 diabetes. Type 2 diabetes and critical illness have phenotypical similarities, such as hyperglycaemia, insulin resistance and systemic inflammation. Previous studies have shown beneficial effects of exogenous glucagon-like peptide (GLP)-1 on glycaemia in critically ill patients, a phenomenon also seen in patients with type 2 diabetes. In this study, we hypothesised that the incretin effect, which is mediated by the incretin hormones GLP-1 and glucose-dependent insulinotropic peptide (GIP), is impaired in critically ill patients.

**Methods:**

The incretin effect (i.e., the relative difference between the insulin response to oral and intravenous glucose administration) was investigated in a cross-sectional case–control study. Eight critically ill patients without diabetes admitted to a mixed intensive care unit and eight healthy control subjects without diabetes, matched at group level by age, sex and body mass index, were included in the study. All subjects underwent an oral glucose tolerance test (OGTT) followed by an intravenous glucose infusion (IVGI) on the next day to mimic the blood glucose profile from the OGTT. Blood glucose, serum insulin, serum C-peptide and plasma levels of GLP-1, GIP, glucagon and proinflammatory cytokines were measured intermittently. The incretin effect was calculated as the increase in insulin secretion during oral versus intravenous glucose administration in six patients. The groups were compared using either Student’s *t* test or a mixed model of repeated measurements.

**Results:**

Blood glucose levels were matched between the OGTT and the IVGI in both groups. Compared with control subjects, proinflammatory cytokines, tumour necrosis factor α and interleukin 6, were higher in patients than in control subjects. The endogenous response of GIP and glucagon, but not GLP-1, to the OGTT was greater in patients. The insulin response to the OGTT did not differ between groups, whereas the insulin response to the IVGI was higher in patients. Consequently, the calculated incretin effect was lower in patients (23 vs. 57 %, *p* = 0.003).

**Conclusions:**

In critically ill patients, the incretin effect was reduced. This resembles previous findings in patients with type 2 diabetes.

**Trial registration:**

ClinicalTrials.gov identifier: NCT01347801. Registered on 2 May 2011.

**Electronic supplementary material:**

The online version of this article (doi:10.1186/s13054-015-1118-z) contains supplementary material, which is available to authorized users.

## Introduction

Elevated blood glucose is often seen in critically ill patients without diabetes [1]. The severity of this hyperglycaemia is correlated to mortality and morbidity in such patients [2], and normoglycaemia obtained by insulin infusion may reduce mortality and morbidity [3, 4]. However, despite the clear importance of hyperglycaemia to these hard endpoints, the pathophysiological basis of hyperglycaemia in this context is not fully understood.

Type 2 diabetes and critical illness have similar characteristics, both presenting with insulin resistance (IR) and systemic inflammation, which contribute to the hyperglycaemic state [5, 6]. In type 2 diabetes, the development of hyperglycaemia is facilitated by pancreatic β-cell dysfunction, including a reduced incretin effect, which is defined as the relative increase in insulin secretion induced by oral versus intravenous glucose administration. This effect is induced largely by the intestinal hormones, glucagon-like peptide (GLP)-1 and glucose-dependent insulinotropic peptide (GIP) [7, 8]. Understanding the incretin effect in critical illness is relevant, given the recent interest in using incretin analogues in the management of hyperglycaemia in the intensive care unit (ICU) [9]. Thus, incretin analogues may be associated with a lower risk of hypoglycaemia [10, 11] than current insulin treatment regimens [12]. However, our understanding of the incretin system in ICU patients is insufficient to recommend routine use of incretin analogues in these patients.

Although type 2 diabetes and critical illness share some phenotypic characteristics, a major difference is that hyperglycaemia in critical illness develops in a matter of days rather than years. Interestingly, acute experimental hyperglycaemia impairs pancreatic β-cell function [13, 14], incretin action [13, 15] and the incretin effect in humans [15]. Additionally, glucose normalisation with insulin treatment also improves β-cell function and the incretin effect in type 2 diabetes [16–18]. Furthermore, clinical studies by our group demonstrate that systemic inflammation induced by tumour necrosis factor (TNF)-α infusion impaired the suppression of GLP-1 on endogenous glucose production [19], but did not change the incretin effect [20], in healthy humans. These findings suggest that the incretin effect may become impaired in critical illness as a consequence of hyperglycaemia rather than inflammation per se. This notion is supported by evidence that the incretin effect is impaired in patients with chronic pancreatitis in the presence of hyperglycaemia only [21]. Consequently, in the present study, we hypothesised that the incretin effect in critically ill patients would be reduced compared with the effect in age- sex- and body mass index (BMI)-matched healthy control subjects without diabetes.

## Material and methods

**Fig. 1:**
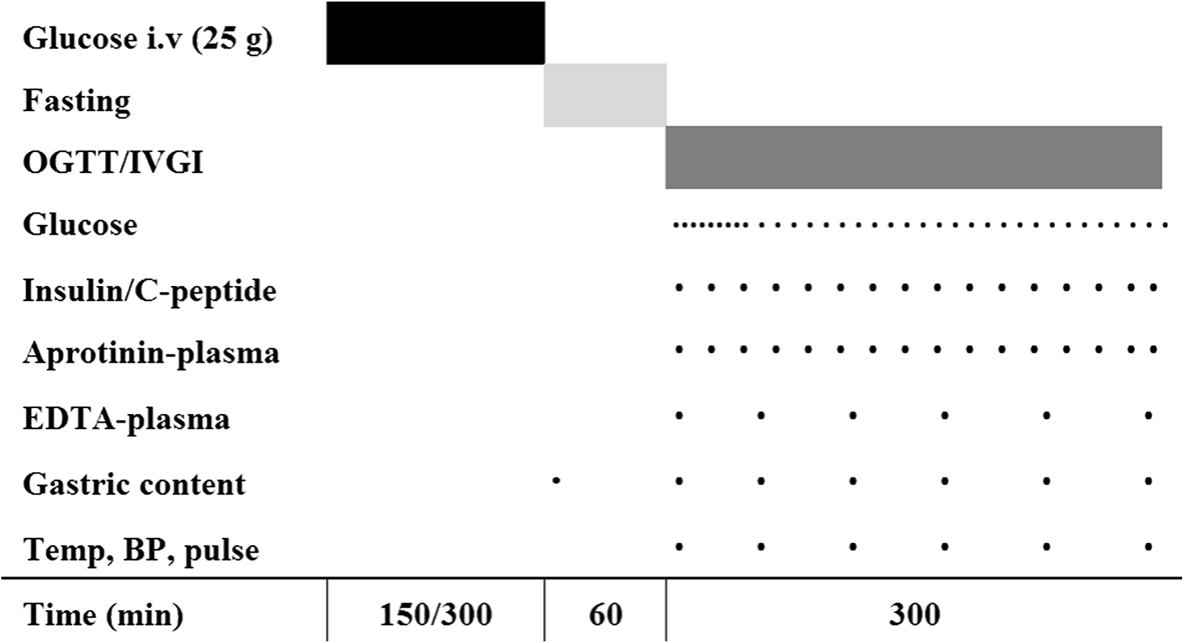
Study design. Glucose (50 mg/ml, 25 g in total) was infused intravenously (i.v.) for 150 minutes in the control subjects and for 300 minutes in the critically ill patients. *Circle* indicates blood sampled for analysis. *Square* indicates a continous intervention. *BP* blood pressure, *EDTA* ethylenediaminetetraacetic acid, *IVGI* intravenous glucose infusion, *OGTT* oral glucose tolerance test

Eight patients admitted to a mixed ICU and eight healthy control subjects without diabetes, who were matched at group level by age, sex and BMI, were included in the study (Fig. 1). The study was conducted from April 2012 to May 2013. Informed consent was obtained from the ICU patients or, when this was not possible, from their next of kin and their general practitioner according to Danish law. The control subjects gave oral and written informed consent to participate. The study was approved by the Scientific Ethics Committee of the Capital Region of Denmark (file number H-3-2009-108), approved by The Danish Data Protection Agency and conducted in accordance with the Declaration of Helsinki.

### Subjects and procedures

#### Critically ill patients

Eight nondiabetic critically ill patients [haemoglobin A1c (HbA1c) <6.5 %] were enrolled in the study no more than 72 h after admission to the ICU. Patients were excluded if they had undergone recent abdominal surgery (except simple appendectomy), had acute or chronic pancreatitis, had a history of bariatric surgery, were aged <18 years, or if informed consent could not be obtained. Information about physical activity level in the 30 days before admission was obtained from the patient’s next of kin and graded on a scale from 1–5, with 1 being no physical activity at all and 5 being vigorous physical activity for more the 4 h per week.

On 2 consecutive days, we performed an oral glucose tolerance test (OGTT; 75 g of glucose in 300 ml of water) and an isoglycaemic intravenous glucose infusion (IVGI) (glucose; Fresenius Kabi, Uppsala, Sweden) using a variable rate designed to match the plasma glucose profile measured during the OGTT. For the OGTT, the glucose solution was injected into the stomach through a nasogastric tube with the patient in an upright position; for the IVGI, glucose was infused through a central venous catheter. Six hours before each of these two procedures, all routine enteral and parenteral nutrition was discontinued. To avoid hypoglycaemia induced by fasting, the patients received a glucose infusion (total 25 g of glucose) for 5 h until 1 h before the OGTT and IVGI protocol was commenced. At this time, the glucose and insulin infusions, if any, were discontinued, and the stomach was emptied via the nasogastric tube. Glucose emptying from the stomach during OGTT was assessed hourly by measuring the gastric content via the gastric tube. Throughout the procedures, blood for glucose analysis was obtained from an arterial catheter located in the radial artery, whereas blood for other analyses was sampled from a central venous catheter through an infusion line separated from the glucose infusion.

#### Healthy control subjects

Eight healthy age-, sex- and BMI-matched control subjects without diabetes (Hb1Ac <6.5 %) were included. Subjects were excluded if they had a history of recent abdominal surgery (except for simple appendectomy), bariatric surgery or cancer or if they had any chronic inflammatory disease. Information about physical activity level in the last 30 days was graded on a scale from 1 to 5, with 1 being no physical activity at all and 5 being vigorous physical activity for more the 4 h per week.

An OGTT and an isoglycaemic IVGI were performed on 2 consecutive days. On each day, subjects reported to the laboratory after an overnight fast, and baseline blood samples were collected from an antecubital venous catheter. For measurement of blood glucose, arterialised blood was collected from a retrograde venous catheter on the dorsum of the hand wrapped in a heating blanket. On both study days, glucose was infused through a cubital venous catheter on the opposite arm to mimic the pretest glucose infusion provided to the critically ill patients. This infusion was limited to a duration of 2½ h (total 25 g of glucose) for logistic reasons, but it was discontinued at 1 h before the OGTT or IVGI, as was the case for the critically ill patients. During the OGTT and IVGI, blood samples were obtained according to the schedule shown in Fig. 1.

### Blood samples

Blood samples from the two groups were collected, stored and analysed in an identical fashion.

#### Blood glucose

Arterial blood samples (in patients) and arterialised venous blood samples (in healthy control subjects) were analysed during the OGTT and IVGI every 5 minutes for the first hour and every 10 minutes for the next hours using a bedside glucose oxidase assay (ABL 725 series; Radiometer, Copenhagen, Denmark).

#### Insulin and C-peptide

Blood was sampled in serum-separating tubes, stored upright at room temperature for 20–40 minutes and centrifuged at 3500 rpm. Serum was removed and stored at 5 °C until samples were analysed by electrochemical enzyme-linked immunosorbent assay (ECLIA) (Roche E-modular; Roche, Basel, Switzerland) within 6 h at the Department of Clinical Biochemistry, Rigshospitalet, Copenhagen, Denmark.

#### GLP-1, GIP and glucagon

Blood was sampled in ethylenediaminetetraacetic acid (EDTA)-coated tubes with aprotinin (Trasylol; Bayer HealthCare, Leverkusen, Germany) 5000 IU/ml blood, immediately centrifuged at 3500 rpm and stored at −80 °C until analysis. Total GLP-1 was analysed using an ECLIA (Meso Scale Discovery, Rockville, MD, USA) with a detection range of 0.98–1000 pg/ml and intraassay variation of 1.1–7.6 %. Total GIP was analysed using an enzyme-linked immunosorbent assay (EMD Millipore, Billerica, MA, USA) with a detection range of 4.2–2000 pg/ml, intraassay variation of 3.0–8.8 % and interassay variation of 1.8–6.1 %. Total glucagon was analysed using a radioimmunoassay (Electra-Box Diagnostica, Copenhagen, Denmark) with a detection range of 18.45–400 pg/ml and intraassay variation of 4.0–6.8 %. Only one assay was used for glucagon measurements. All samples were analysed in duplicates on stored plasma, and the mean value was used for further analysis.

#### IL-6 and TNF-α

Blood was sampled in EDTA-coated tubes, immediately centrifuged at 3500 rpm and stored at −80 °C until analysis. Interleukin (IL)-6 and TNF-α were analysed using ECLIA (Meso Scale Discovery). For IL-6, the detection range was 1.58–488 pg/ml, intraassay variation was 5.2–7.3 % and interassay variation was 4.2–5.5 %. For TNF-α, the detection range was 0.69–248 pg/ml, intraassay variation was 6.1–10.1 % and interassay variation 6.2–7.2 %. All samples were analysed in duplicates on stored plasma, and the mean value was used for further analysis.

In two of the patients, it was not possible to perform the IVGI. One patient did not want to participate in the second study day, and fasting was not accomplished according to the protocol in the other patient. In addition, two critically ill patients did not complete the IVGI to 180 minutes, one due to transfer to an acute percutaneous coronary intervention and the other due to termination of treatment (at 80 min 120 min, respectively). Thus, we present OGTT data for eight patients and eight control subjects and IVGI data for six patients and eight control subjects. Data from the two patients who did not complete the IVGI to 180 minutes was extrapolated as described below.

### Calculations

The incretin effect was calculated as incretin effect = [incremental area under the curve (iAUC) insulin OGTT − iAUC insulin IVGI]/iAUC insulin OGTT [8].

The Matsuda index, a marker of insulin sensitivity, was calculated as follows [22]:

$$ \mathrm{Matsuda}\ \mathrm{index}=10,000/\sqrt{\left(\left(\mathrm{baseline}\ \mathrm{glucose}*\mathrm{mean}\ \mathrm{glucose}\right)*\left(\mathrm{baseline}\ \mathrm{insulin}\ *\ \mathrm{mean}\ \mathrm{insulin}\right)\right)} $$

The homeostatic model assessment (HOMA)-IR, a marker of IR, was calculated as HOMA-IR = (fasting glucose × fasting insulin)/22.5 [23].

HOMA-β, a marker of β-cell function, was calculated as HOMA‐β = (20 × fasting insulin)/(fasting glucose − 3.5) [23].

### Statistics

The statistical analyses were performed using SAS 9.3 software (SAS Institute, Cary, NC, USA). All statistical analyses were based on iAUCs during the OGTT and the IVGI, except for Matsuda index, HOMA-IR and HOMA-β. iAUC was calculated using the trapezoidal rule.

Group differences in the incretin effect, the Matsuda index, HOMA-IR, and HOMA-β were evaluated using unpaired *t* tests. Group and trial differences in iAUCs for glucose, insulin, C-peptide, glucagon, GLP-1, GIP, TNF-α and IL-6 were evaluated using a mixed model of repeated measurements adjusted for difference in variation in the data between the groups. *p* < 0.05 was considered significant. If a better fit of normal distribution was obtained by log transformation, this was performed before the statistical analysis. Since the number of patients was small, we also performed nonparametric testing of these variables.

In the text and figures, the incretin effect, Matsuda index, HOMA-IR, HOMA-β and iAUC of glucose are given as mean and 95 % confidence interval (CI), whereas iAUCs of insulin, C-peptide, glucagon, GLP-1, GIP, TNF-α and IL-6 are given as geometric mean and 95 % CI. The changes over time during OGTT and IVGI are given as mean or geometric mean and 95 % CI because the statistics of these measurements were performed with the calculated iAUCs.

The number of participants was determined by a power calculation based on data from a comparable study [20] (G*Power 3.0 software) (α = 0.05 and β = 0.2).

Missing data were imputed either by using the average of the value recorded one time point before and one time point after the missing value or, if the data were missing in the beginning or at the end of the OGTT or IVGI, by multiplying the last available value by the average percentage change in the other subjects in the group at that time point. An overview of the missing data is provided as Additional file 1.

Blood samples were obtained for 300 minutes after glucose ingestion. Due to missing IVGI data from the two patients who only partly finished the IVGI, we analysed only the data from the first 180 minutes in order to avoid too many imputations.

## Results

### Subject characteristics

Table 1 summarises the characteristics of the groups. The patients and the control subjects were matched with regard to mean age, sex distribution and mean BMI. Eight patients were included in the study. Individual details on the eight patients are given in Table 2. Three of the patients died in the ICU (38 % ICU mortality). The OGTT and IVGI were performed without any complications. During the OGTT, as evaluated by aspiration of gastric contents, the stomach was empty after 60 minutes in two patients, after 120 minutes in one patient and after 180 minutes in three patients. In two patients, the stomach was still not empty after 180 minutes.

**Table 1:** Subject characteristics

	Patients (n = 8)	Control subjects (n = 8)
Mean/median age (yr)	68.8/69.5	66.1/66.5
	(64.5–73.5)	(64.5–68.5)
Mean/median BMI (kg/m^2^)	25.0/24.0	25.2/24.7
	(21.0–28.0)	(22.0–27.4)
Male/female distribution	4/4	4/4
Mean/median HbA1c (%)	6.1/6.2	5.6/5.6
	(5.9–6.4)	(5.5–5.7)
Mean/median physical activity level (1–5)	2.3/2.0	3.5/4.0
	(1.0–3.0)	(3.0–4.0)

*BMI* body mass index, *HbA1c* haemoglobin A1c

Except for male/female distribution, values are mean/median (interquartile range)

The data on physical activity level are from seven critically ill patients and eight control subjects

**Table 2:** Individual data on the eight patients included in the study

Patient	Reason for admission to the ICU	Sex	APACHE II	Data from IVGI	OGTT on day after admission	Discharge destination	Days in ICU	Ventilator OGTT/IVGI	Mean/median (interquartile range) blood glucose 36 h before OGTT (mmol/L)	Total dose of insulin 36 h before OGTT (IU)	Total dose of steroid^a^ in ICU before OGTT (mg)	Dose of steroid^a^ OGTT/IVGI (mg/day)	Dose of NE OGTT/IVGI (mg/kg/day)
1	Sepsis (pneumonia)	Male	27	No	2	Ward	2.8	Yes	8.2/7.7(6.4–8.7)	None	18.7	3.7	None/
2	Sepsis (pneumonia)	Female	34	Yes	2	Deceased	4.4	Yes/Yes	9.0/7.7(6.4–10.3)	2	None	None/11.2	0.53/1.15
3	Sepsis (diverticulitis)	Male	23	No	2	Ward	9.0	No	10.4/9.7(9.1–11.3)	None	None	None/	None/
4	Cardiogenic shock	Female	25	Yes	2	ICU at another hospital	2.0	No/No	11.9/11.3(10.1–12.1)	None	5.6	5.6/None	None/None
5	Hypothermia after cardiac arrest	Male	41	Yes	4	Deceased	5.2	Yes/Yes	8.1/8.4(7.9–9.4)	63	None	None/None	None/None
6	Sepsis (pneumonia)	Female	45	Yes	3	Deceased	4.1	Yes/Yes	8.2/7.4(6.6–8.5)	None	18.8	None/None	0.04^b^/0.59
7	Sepsis (pneumonia)	Male	31	Yes	2	Ward	12.7	No/Yes	7.2/7.6(7.1–8.7)	None	None	None/None	None/None
8	Sepsis (pneumonia)	Female	24	Yes	3	Ward	14.4	Yes/Yes	9.5/9.4 (7.9–10.9)	48	11.2	5.6/5.6	0.04/0.20
Mean/median (interquartile range)			31/28 (24–38)				6.8/4.8 (3.5–10.8)		9.1/8.6 (8.1–9.9)	14.1/0.0 (0.0–25.0)	6.8/2.8 (0.0–15.0)	1.8/0.0 (0.0–4.7)/2.8/0.0 (0.0–5.6)	0.08/0.00 (0.00–0.04)/0.32/0.10 (0.0–0.59)

*APACHE* Acute Physiology and Chronic Health Evaluation, *ICU* intensive care unit, *IVGI* intravenous glucose infusion, *NE* norepinephrine, *OGTT* oral glucose tolerance test, *IU* International Units

Patients on the ventilator included intubated patients and patients receiving noninvasive ventilation.

^a^Dose equipotent to dexamethasone

^b^This patients also received 22 mg/kg/day of dopamine infusion on this day

### Blood glucose

**Fig. 2:**
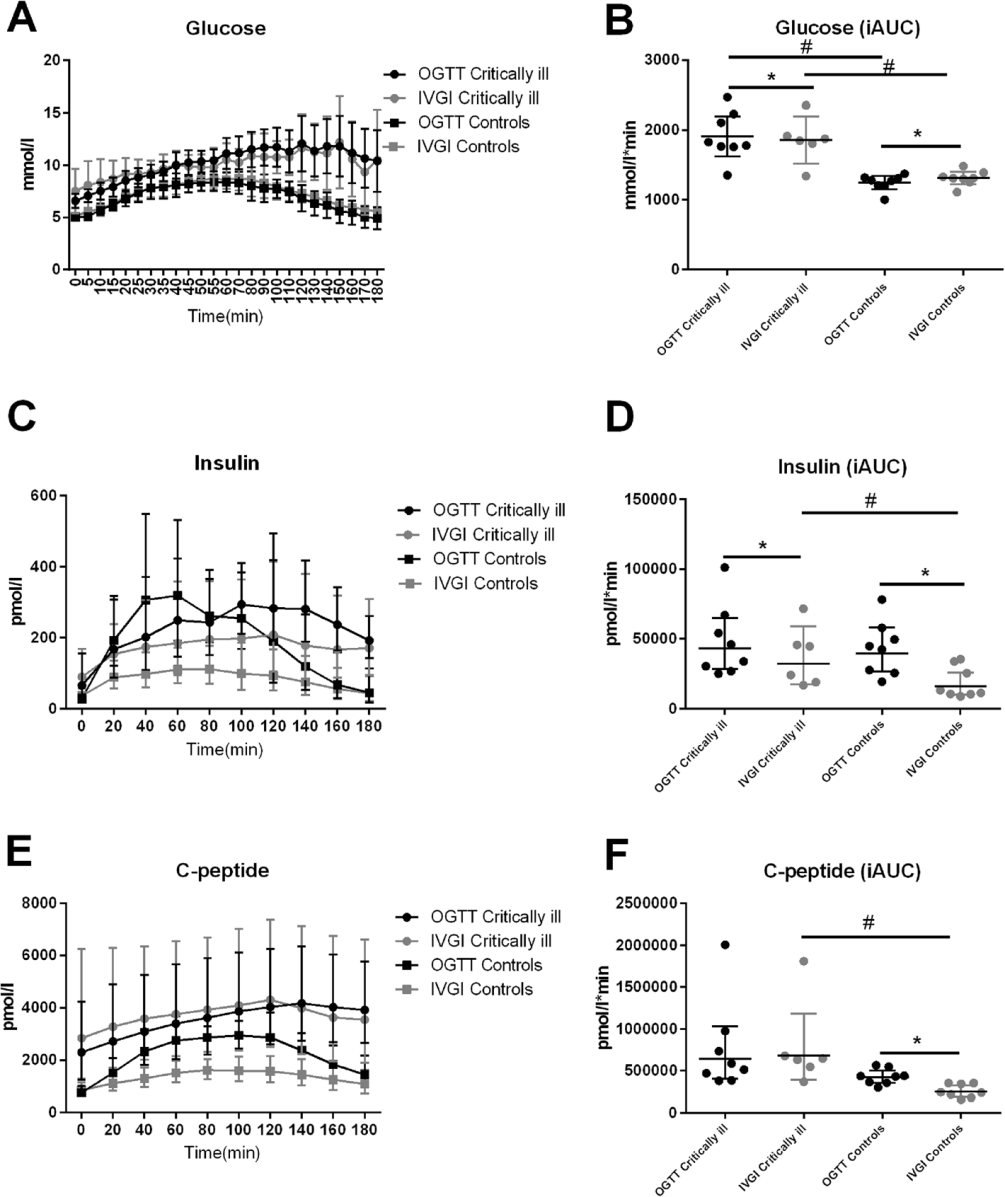
Glucose, insulin and C-peptide. Mean blood glucose (**a**) was measured every 5 minutes for the first 60 minutes and every 10 minutes for the next 120 minutes after glucose ingestion. Geometric mean insulin (**c**) and geometric mean serum C-peptide (**e**) [with 95 % confidence interval (CI)] were measured every 20 minutes for 180 minutes after glucose ingestion. Mean iAUC of blood glucose (**b**), geometric mean iAUC of serum insulin (**d**) and geometric iAUC mean of serum C-peptide (**f**) (95 % CI). *Significant difference between OGTT and IVGI; ^#^significant difference between groups. *iAUC* incremental area under the curve, *IVGI* intravenous glucose infusion, *OGTT* oral glucose tolerance test

Mean blood glucose was higher in patients than in control subjects during the OGTT (*p* = 0.007) (Fig. 2). Compared with values during the OGTT, blood glucose during the IVGI was significantly higher in control subjects (*p* = 0.0158) and significantly lower in patients (*p* = 0.035).

### Insulin and C-peptide

As expected, insulin increased during both the OGTT and the IVGI and was higher during the OGTT than during the IVGI in both groups (*p* = 0.038 for patients, *p* = 0.0001 for control subjects), corroborating the presence of an incretin effect. Furthermore, insulin was higher in patients than in control subjects during the IVGI (*p* = 0.045) but not during the OGTT.

Not surprisingly given the insulin response, C-peptide also increased in both groups and was higher in patients than in control subjects during the IVGI (*p* = 0.004) but not during the OGTT. Interestingly, however, the serum concentration of C-peptide did not differ between the OGTT and the IVGI in patients, whereas it was higher during the OGTT than during the IVGI in the control subjects (*p* < 0.0001).

### Incretin hormones

**Fig. 3:**
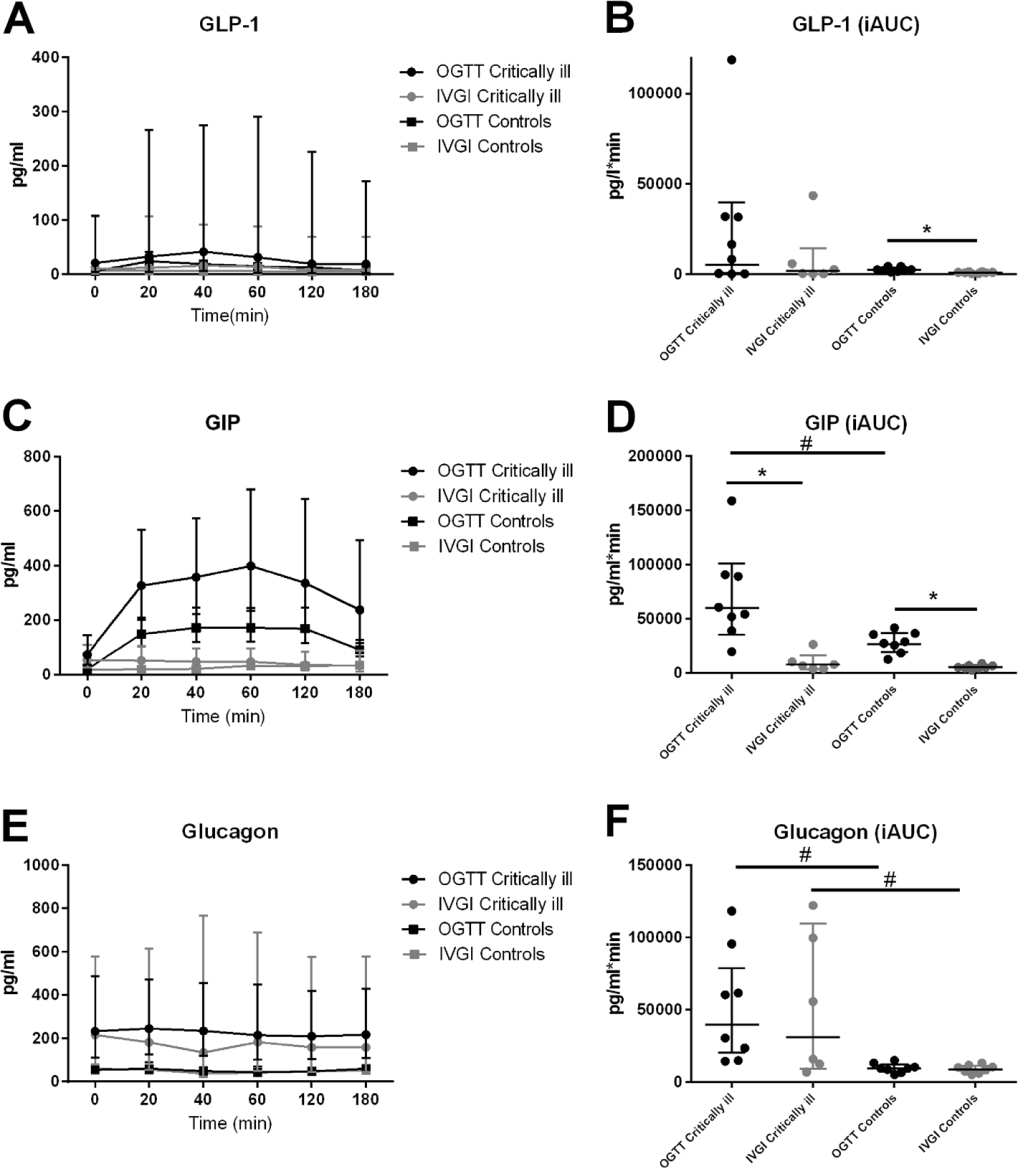
The incretin hormones and glucagon. Geometric mean plasma GLP-1 (**a**), geometric mean plasma GIP (**c**) and geometric mean plasma glucagon (**e**) measured 0, 20, 40, 60, 120 and 180 minutes after glucose ingestion, all with 95 % confidence intervals (CIs). Geometric mean iAUC of plasma GLP-1 (**b**), geometric mean iAUC of plasma GIP (**d**) and geometric mean iAUC of plasma glucagon (**f**) (with 95 % CI). *Significant difference between OGTT and IVGI; ^#^significant difference between groups. *GIP* glucose-dependent insulinotropic peptide, *GLP* glucagon-like peptide, *iAUC* incremental area under the curve, *IVGI* intravenous glucose infusion, *OGTT* oral glucose tolerance test

Plasma levels of both GIP and GLP-1 rose during the OGTT in both groups. GLP-1 did not differ between the groups during the OGTT or the IVGI. However, GLP-1 was higher during the OGTT than during the IVGI in control subjects (*p* = 0.0006) but not in patients (Fig. 3). GIP was higher in patients than in control subjects during the OGTT (*p* < 0.0001) but not during the IVGI. Levels were higher during the OGTT than during the IVGI both in control subjects and in patients.

### Glucagon

Plasma glucagon was higher in patients than in control subjects (*p* = 0.001 for OGTT, *p* = 0.046 for IVGT) but did not change during the OGTT or the IVGI in either group (Fig. 3).

### Cytokines

**Fig. 4:**
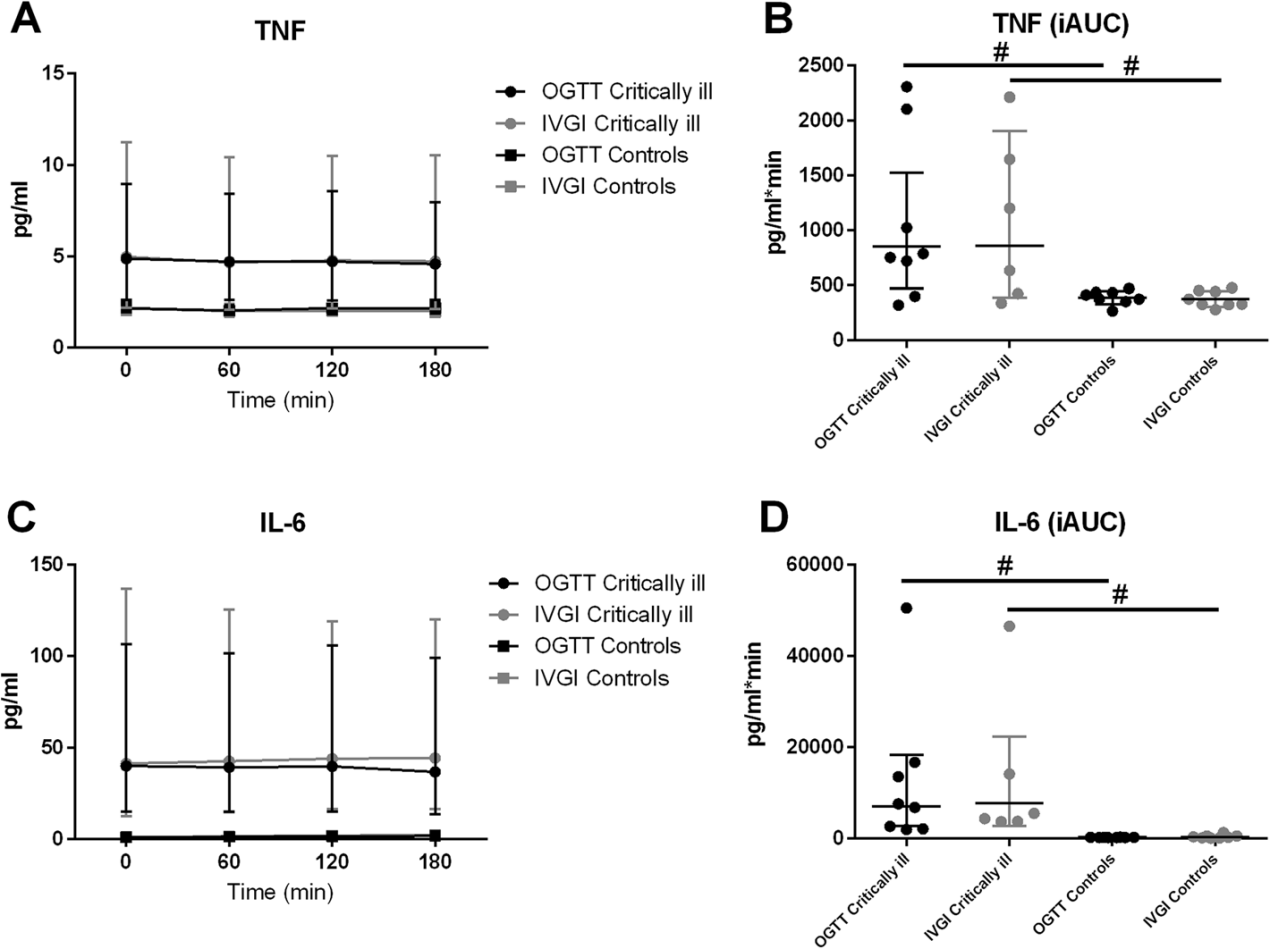
TNF-α and IL-6. Geometric mean plasma TNF-α (**a**) and geometric mean plasma IL-6 (**c**) measured at baseline and hourly after glucose ingestion, both with 95 % confidence intervals (CI). Geometric mean iAUC of plasma TNF-α (**b**) and geometric mean iAUC of plasma IL-6 (**d**) with 95 % CI. ^#^Significant difference between groups. *iAUC* incremental area under the curve, *IL* interleukin, *IVGI* intravenous glucose infusion, *OGTT* oral glucose tolerance test, *TNF* tumour necrosis factor

Plasma levels of the proinflammatory cytokines TNF-α and IL-6 were higher in the patients than in control subjects (TNF-α, *p* = 0.015 for OGTT and *p* = 0.042 for IVGI; IL-6, *p* < 0.0001 for OGTT and *p* < 0.0001 for IVGI), indicating the presence of systemic inflammation. No difference occurred during the OGTT and the IVGI in either group (Fig. 4).

### Incretin effect

**Fig. 5:**
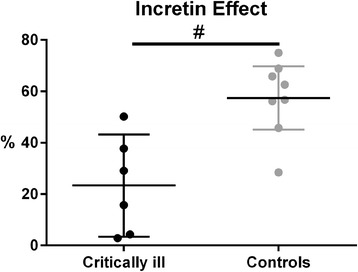
Mean incretin effect with 95 % confidence interval. ^#^Significant difference between groups

The incretin effect was less in patients than in control subjects [23 % (3–43 %) vs. 57 % (45–70 %); *p* = 0.003] (Fig. 5).

### Insulin sensitivity and fasting insulin resistance

Insulin sensitivity as measured by the Matsuda index tended to be lower in patients than in control subjects [2.08 (3.94–7.46) vs. 7.76 (5.09–11.8); *p* = 0.056). Also, IR as evaluated by HOMA-IR on the OGTT day was higher in patients than in control subjects [2.73 (1.1–6.7) vs. 0.87 (0.53–1.44); *p* = 0.021].

### Fasting β-cell function

There was no difference in HOMA-β between the groups [62.5 (27.1–218.3) in patients vs. 55.0 (33.3–90.9) in control subjects].

### Results of nonparametric testing

Because of the small number of subjects, we performed additional nonparametric tests of data. This rendered nonsignificant the difference in blood glucose between the OGTT and the IVGI in patients (*p* = 0.0345 in mixed model, *p* = 0.0625 by Wilcoxon test), as well as the difference in serum insulin during IVGI between groups (*p* = 0.045 in mixed model, *p* = 0.0593 by Mann–Whitney *U* test). The significance of the remaining analyses remained unchanged, regardless of whether a parametric or nonparametric method was used.

## Discussion

Despite the well-known association between IR, hyperglycaemia and a poor outcome in critically ill patients, the optimal treatment remains elusive. Thus, although van den Berghe and coworkers achieved a much improved survival in different intensive care populations with tight glycaemic control using insulin [3, 4], more recent studies of insulin treatment in these patients have shown no such benefit [24, 25]. Whether this is due to a clinically important increase in hypoglycaemia with insulin or because hyperglycaemia is an epiphenomenon [26] rather than a mediator of a poor outcome, these discrepancies indicate a need to look beyond the hyperglycaemia itself and explore the related pathophysiology. In the present study, while confirming the previously reported presence of hyperglycaemia and IR in critically ill patients admitted to a multidisciplinary ICU, the major finding was an impaired incretin effect. Although researchers in previous studies have investigated plasma levels of incretin hormones, fasting and nonfasting, in critically ill patients [27–30], this study is, to our knowledge, the first investigation of the secretion of incretin hormones in response to a standardised stimulus. The incretins, and in particular GLP-1, have gained much interest during the last few decades as a novel treatment paradigm for type 2 diabetes [31]. This disease is associated with a marked reduction in the incretin effect (i.e., the physiologically increased insulin secretion that occurs in response to enteral compared with intravenous administration of nutrients, and which is due to the secretion of incretin hormones by intestinal K and L cells) [7]. The results of the present study indicate that a similar reduction is present in critically ill patients.

Insulin levels were similar in patients and control subjects during the OGTT despite the presence of much higher blood glucose levels in critically ill patients throughout the study. In fact, patients’ blood glucose levels failed to return to baseline at 180 minutes after glucose ingestion. The maximal insulin secretory response of the β cell depends on hyperglycaemia and exposure to arginine. Although we did not measure plasma arginine, levels are reported to be elevated in critically ill patients in the first week of admission to the ICU [32, 33]. Adding the level of hyperglycaemia before the OGTT, it is possible that the β cells were acting towards the top of their secretory capacity during the OGTT, secreting the insulin available. The fact that there was no significant difference in C-peptide concentrations during the OGTT and the IVGI in the patients supports this argument. However, the increase in serum insulin and in C-peptide, albeit not to the same extent as insulin, during the OGTT in patients could indicate a secretory reserve, although large variation in data needs to be taken into consideration when interpreting the difference in C-peptide and insulin concentrations.

Both incretin hormones increased during the OGTT. Furthermore, GIP, but not GLP-1, increased more in the patients than in the control subjects during the OGTT. This indicates that L- and K-cell function was preserved in the patients, even though the resulting levels of GIP and GLP-1 were insufficient to elicit an appropriate insulin-secretory response in terms of controlling hyperglycaemia. In healthy volunteers, hyperglycaemia induced by glucose infusion to a level of 10 mmol/L for 24 h reduces sensitivity to GLP-1 in the β cell [13]. Clinical studies have shown that infusion of exogenous GLP-1 decreases the glycaemic response to enteral nutrition in critically ill patients, including those with preexisting type 2 diabetes [34, 35]; however, the sensitivity to exogenous GIP is impaired, as has also been reported in type 2 diabetes [30]. We speculate that the elevated levels of GLP-1 and GIP, in combination with the hyperglycaemia in the patients, indicate a reduced sensitivity of the β cell to the insulinotropic effects of the incretins. This suggestion is supported by in vitro experiments [36].

Glucocorticoids induce hyperglycaemia, decrease the incretin effect and increase the response of both GLP-1 and GIP [37], and reduce the insulinotropic properties of GLP-1 and GIP in healthy volunteers [38]. Glucocorticoid treatment was used in five of eight patients. This, as well as elevated level of endogenous cortisol, which is frequently induced by critical illness [39], may therefore contribute to the present findings. Plasma levels of GLP-1, GIP and glucagon were elevated at baseline in patients (*p* < 0.05). GLP-1 and glucagon are derived from the proglucagon molecule, which is produced in the L cell in the small intestine [7, 40]. This indicates that the L and K cells were continuously stimulated in these patients, either because enterally administered nutrients were still present in the distal part of the small intestine after 6 h of fasting or due to the action of another local stimulant, which could be IL-6 [27].

### Cytokines and insulin resistance

Critically ill patients exhibit systemic inflammation. However, systemic inflammation induced by TNF-α infusion in healthy human volunteers does not affect glucose-stimulated insulin secretion [41] or the incretin effect [20], despite inducing IR and reducing the suppressive effect of GLP-1 on endogenous glucose production [19, 20]. On one hand, in cell studies, TNF-α alone does not impair insulin secretion, but, when combined with other proinflammatory cytokines, it induces β-cell dysfunction [42]. On the other hand, the patients had IR as evaluated by HOMA-IR. Since TNF-α induces IR in skeletal muscle [43], systemic elevations of TNF-α in critically ill patients may cause IR. As an alternative explanation, physical activity levels were lower in the patients than in control subjects, which could also contribute to IR in this group [44].

Gastric emptying affects the magnitude of the incretin effect in healthy subjects and in patients with type 2 diabetes [45], and prolonged gastric emptying was observed in some of the patients, even though this was evaluated rather crudely by assessing the remaining gastric fluid contents by aspiration. Thus, the reduced incretin effect may have been due to or enhanced by the presence of delayed gastric emptying in our patients. Furthermore, five of eight patients received glucocorticoid treatment in the ICU in the days before and during the test days; this is also known to reduce the incretin effect [37].

### Limitations

We included only a small number of patients, which was primarily due to the very complex setup in the setting of serious critical illness with the potential for dramatic changes in clinical presentation. The low number of patients and the fact that some patients were on steroids limit the generalisability of the findings. Extrapolation of data from the IVGI in two patients potentially reduces the variability in the data. With regard to glycaemic control, preexisting hyperglycaemia may have contributed to the impaired incretin effect [46]. The method of choice to investigate the incretin effect is to conduct the OGTT and the IVGI on 2 consecutive days [47]. Although we conducted the studies in the early phase of admission to the ICU, we cannot rule out the possibility that some of the patients were already in the recovery phase of their critical illness. Furthermore, because critical illness is a dynamic condition, 2 days might not be perfectly comparable with regard to severity of disease. The variations in BMI and age among the patients were slightly greater than in the control subjects; also, the mean and median age was higher in the patients. However, we believe that the matching was sufficient. Due to logistics, the study protocols in the two groups were not completely identical with regard to the duration of fasting and the duration of pretest glucose infusion. Additionally, the site of blood glucose measurements was arterial blood in the patients and arterialised venous blood in control subjects. However, we do not believe that these small differences affected the outcomes significantly.

In the patients, the iAUC of glucose during the OGTT was slightly higher than that during the IVGI, whereas it was slightly lower during the OGTT in control subjects. These differences may have led to an overestimation of the incretin effect in patients while underestimating it in control subjects. However, we believe that the large difference in the incretin effect is very unlikely to be due to the very small unintended differences in blood glucose between trials. GLP-1 and GIP levels were analysed in plasma samples obtained in collecting tubes without dipeptidyl peptidase (DPP)-4 inhibitor in both groups. Despite the fact that the blood was obtained in iced tubes and the samples were centrifuged rapidly, there is a possibility that some of the GLP-1 and GIP was degraded by the enzyme DPP-4 in the tubes before centrifugation, leading to potentially lower measured plasma levels in both groups. HbA1c is slightly higher in patients, which we believe reflects hyperglycaemia related to critical illness.

Gastric emptying was evaluated semiquantitatively by the amount of fluid aspirated from the gastric tube. This is not the gold standard of measuring gastric emptying, and we confirmed the position of the gastric tube only by aspiration of gastric content. Furthermore, gastric emptying was not measured in the control subjects. Imputation of missing data to allow us to calculate the iAUC was done mainly in the patients. This led to a reduction of the variance in that group, which is a limitation in the data. Use of a dual or triple glucose tracer approach would have allowed determination of hepatic glucose production during OGTT; however, we did find significantly higher glucagon levels in the patients, which might indicate a higher rate of hepatic glycogenolysis in this group.

## Conclusions

This study indicates that a reduced incretin effect accompanies IR and hyperglycaemia in critically ill patients. The results indicate preserved L- and K-cell function in critically ill patients. Thus, the increase in GIP and GLP-1 secretion during the OGTT was not sufficient to reduce blood glucose to normal levels.

We suggest that while IR in critically ill patients is a consequence of systemic inflammation mediated by TNF-α per se, the impaired incretin effect is not directly related to inflammation; it more likely occurs secondary to the development of hyperglycaemia. Delayed gastric emptying and corticosteroid treatment in critically ill patients may further reduce the incretin effect. This may have implications for the treatment of hyperglycaemia in ICU patients in the future.

## Key messages

- The incretin effect may be reduced in critically ill patients.
- The function of intestinal L cells and K cells seems to be preserved in critically ill patients.
- The pancreatic β cell may be resistant to the insulinotropic effects of the incretin hormones in critically ill patients.

## Additional file

Additional file 1:
**Overview of missing data in patients and control subjects.** (DOCX 17 kb)

## Abbreviations

APACHE: Acute Physiology and Chronic Health Evaluation
BMI: Body mass index
DPP-4: Dipeptidyl peptidase 4
ECLIA: Electrochemical enzyme-linked immunosorbent assay
EDTA: Ethylenediaminetetraacetic acid
GIP: Glucose-dependent insulinotropic peptide
GLP: Glucagon-like peptide
HbA1c: Haemoglobin A1c
HOMA: Homeostatic model assessment
iAUC: Incremental area under the curve
ICU: Intensive care unit
IL: Interleukin
IR: Insulin resistance
IVGI: Intravenous glucose infusion
NE: Norepinephrine
OGTT: Oral glucose tolerance test
TNF: Tumour necrosis factor
