# Supplementary material for: The incretin effect in critically ill patients: a case–control study
Source: Crit Care. 2015 Nov 16;19:402. doi: 10.1186/s13054-015-1118-z (PMC4645481; doi:10.1186/s13054-015-1118-z)
Supplement: Additional file 1: — Overview of missing data in patients and control subjects. (DOCX 17 kb) [file 13054_2015_1118_MOESM1_ESM.docx]

**Overview of missing data in patients and controls**

| **Patients** | **Glucose**  **OGTT** | **Glucose**  **IVGI** | **Insulin**  **OGTT** | **Insulin**  **IVGI** | **C-peptide**  **OGTT** | **C-peptide**  **IVGI** | **GLP-1**  **OGTT** | **GLP-1**  **IVGI** | **GIP**  **OGTT** | **GIP**  **IVGI** | **Glucagon**  **OGTT** | **Glucagon**  **IVGI** | **TNF**  **OGTT** | **TNF**  **IVGI** | **IL-6**  **OGTT** | **IL-6**  **IVGI** |
| --- | --- | --- | --- | --- | --- | --- | --- | --- | --- | --- | --- | --- | --- | --- | --- | --- |
| ***No. of possible measurements*** | ***25*** | ***25*** | ***10*** | ***10*** | ***10*** | ***10*** | ***6*** | ***6*** | ***6*** | ***6*** | ***6*** | ***6*** | ***4*** | ***4*** | ***4*** | ***4*** |
| **1** | 0 |  | 0 |  | 0 |  | 0 |  | 0 |  | 0 |  | 0 |  | 0 |  |
| **2** | 0 | 0 | 0 | 0 | 0 | 0 | 0 | 0 | 0 | 0 | 0 | 0 | 0 | 0 | 0 | 0 |
| **3** | 2 |  | 0 |  | 0 |  | 0 |  | 0 |  | 0 |  | 0 |  | 0 |  |
| **4** | 0 | 10 | 0 | 5 | 0 | 5 | 0 | 2 | 0 | 2 | 0 | 2 | 0 | 2 | 0 | 2 |
| **5** | 0 | 6 | 0 | 4 | 0 | 4 | 0 | 1 | 0 | 1 | 0 | 1 | 0 | 1 | 0 | 1 |
| **6** | 0 | 0 | 0 | 0 | 0 | 0 | 0 | 0 | 0 | 0 | 0 | 0 | 0 | 0 | 0 | 0 |
| **7** | 0 | 0 | 0 | 0 | 0 | 0 | 0 | 0 | 0 | 0 | 0 | 0 | 0 | 0 | 0 | 0 |
| **8** | 0 | 0 | 0 | 0 | 0 | 0 | 0 | 0 | 0 | 0 | 0 | 0 | 0 | 0 | 0 | 0 |
| **Total no. of missing data in patients** | 2/200 | 16/150 | 0/80 | 9/60 | 0/80 | 9/60 | 0/48 | 3/36 | 0/48 | 3/36 | 0/48 | 3/36 | 0/32 | 3/24 | 0/32 | 3/24 |
|  | | | | | | | | | | | | | | | | |
| **Controls** | **Glucose**  **OGTT** | **Glucose**  **IVGI** | **Insulin**  **OGTT** | **Insulin**  **IVGI** | **C-peptide**  **OGTT** | **C-peptide**  **IVGI** | **GLP-1**  **OGTT** | **GLP-1**  **IVGI** | **GIP**  **OGTT** | **GIP**  **IVGI** | **Glucagon**  **OGTT** | **Glucagon**  **IVGI** | **TNF**  **OGTT** | **TNF**  **IVGI** | **IL-6**  **OGTT** | **IL-6**  **IVGI** |
| ***No. of possible measurements*** | ***25*** | ***25*** | ***10*** | ***10*** | ***10*** | ***10*** | ***6*** | ***6*** | ***6*** | ***6*** | ***6*** | ***6*** | ***4*** | ***4*** | ***4*** | ***4*** |
| **1** | 0 | 0 | 0 | 0 | 0 | 0 | 0 | 0 | 0 | 0 | 0 | 0 | 0 | 0 | 0 | 0 |
| **2** | 0 | 0 | 0 | 0 | 0 | 0 | 0 | 0 | 0 | 0 | 0 | 0 | 0 | 0 | 0 | 0 |
| **3** | 0 | 0 | 0 | 0 | 0 | 0 | 0 | 0 | 0 | 0 | 0 | 0 | 0 | 0 | 0 | 0 |
| **4** | 0 | 0 | 0 | 0 | 0 | 0 | 0 | 0 | 0 | 0 | 0 | 0 | 0 | 0 | 0 | 0 |
| **5** | 0 | 0 | 0 | 0 | 0 | 0 | 0 | 0 | 0 | 0 | 0 | 0 | 0 | 0 | 0 | 0 |
| **6** | 0 | 0 | 0 | 0 | 0 | 0 | 0 | 0 | 0 | 0 | 0 | 0 | 0 | 0 | 0 | 0 |
| **7** | 0 | 0 | 1 | 0 | 1 | 0 | 0 | 0 | 0 | 0 | 0 | 0 | 0 | 0 | 0 | 0 |
| **8** | 0 | 0 | 0 | 0 | 0 | 0 | 0 | 0 | 0 | 0 | 0 | 0 | 0 | 0 | 0 | 0 |
| **Total no. of missing data in controls** | 0/200 | 0/200 | 1/80 | 0/80 | 1/80 | 0/80 | 0/48 | 0/48 | 0/48 | 0/48 | 0/48 | 0/48 | 0/32 | 0/32 | 0/32 | 0/32 |
|  |  |  |  |  |  |  |  |  |  |  |  |  |  |  |  |  |
| **Total no. of missing data** | 2/400 | 16/350 | 1/160 | 9/140 | 1/160 | 9/140 | 0/96 | 3/84 | 0/96 | 3/84 | 0/96 | 3/84 | 0/64 | 3/56 | 0/64 | 3/56 |
|  |  |  |  |  |  |  |  |  |  |  |  |  |  |  |  |  |
